# Whole-Body Computed Tomography (WBCT) for the initial assessment of trauma patients: a retrospective observational study

**DOI:** 10.1007/s10140-026-02488-w

**Published:** 2026-05-11

**Authors:** Abdelaziz Hammo, Rabab Abdelrahman, Husham Abdelrahman, Tarik Abulkhair, Mohamed N. Ahmed, Ayman El-Menyar, Naushad A. Khan, Mohammad Asim, Sandro Rizoli, Hassan Al-Thani

**Affiliations:** 1 Department of Surgery, Trauma Surgery, Hamad Medical Corporation, Doha, Qatar; 2 Department of Surgery, Qatar University Medical College, Doha, Qatar; 3 Department of Radiology, Hamad Medical Corporation, Doha, Qatar; 4 Department of Clinical Medicine, Weill Cornell Medicine, Doha, Qatar

**Keywords:** Whole-body computed tomography, Polytrauma, Severity of injury, Outcomes, Pan CT scan

## Abstract

**Background:**

Clinicians assessing trauma patients are routinely faced with the critical question of when to pursue whole-body computed tomography (WBCT) and when to withhold it to limit unnecessary exposure and resource use. Despite its widespread integration into early trauma care, there remains uncertainty about the best approach for selecting patients for WBCT, particularly given the implications of its findings. We aimed to evaluate the clinical characteristics and outcomes of trauma patients with positive (pWBCT) versus negative (nWBCT) findings.

**Methods:**

A retrospective study of adult trauma patients who underwent WBCT imaging in 2021 and 2022 was conducted. Patients were stratified into pWBCT; at least one acute traumatic injury identified on scan from head to pelvis or nWBCT; no acute traumatic injury detected on scan from head to pelvis. Clinical characteristics, mechanisms of injury, injury severity, Trauma activation level, and outcomes were analyzed. Multivariable logistic regression analysis was performed to identify independent predictors of pWBCT at presentation.

**Results:**

A total of 2,555 patients were included (76.8% pWBCT and 23.2% nWBCT). pWBCT was associated with significantly higher rates of Level 1 trauma activations (20.5% vs 7.8%, *p* = 0.001), higher mean Injury Severity Score (ISS) (15.1 ± 9.9 vs 6.4 ± 4.1; *p* = 0.001), Shock Index (0.72 ± 0.24 vs. 0.67 ± 0.16; *p* = 0.001), and lower Trauma Score and Injury Severity Score (TRISS) (0.932 ± 0.15 vs 0.990 ± 0.02; *p* = 0.001). Patients with pWBCT required significantly higher rates of intubation, exploratory laparotomy, and massive blood transfusion. This group also had increased ICU admission, longer durations of mechanical ventilation, and higher mortality compared with patients with nWBCT (*p* < 0.001). Multivariable logistic regression analysis demonstrated that age (aOR 1.013; 95% CI 1.004—1.022; *p* = 0.003), male gender (aOR 0.687; 95% CI 0.475—0.995; *p* = 0.047), shock index (aOR 1.990; 95% CI 1.045—3.789; *p* = 0.036), lower GCS at ED (aOR 0.852; 95% CI 0.804—0.904; *p* = 0.001), traffic related incidents (aOR 1.464; 95% CI 1.065—2.012; *p* = 0.019), falls from height (aOR 2.545; 95% CI 1.750—3.701; *p* = 0.001), pedestrian crashes (aOR 1.620; 95% CI 1.013—2.588; *p* = 0.044), and a positive FAST (aOR 5.977; 95% CI 2.149—16.623; *p* = 0.001) were independent predictors of pWBCT.

**Conclusion:**

Patients with pWBCT form a high-risk population with greater injury severity, physiological instability, and intervention needs. The proportion of negative scans highlights potential overutilization. Identifying predictors of positive findings may refine imaging decisions, improve resource use, and support risk-stratified strategies to reduce unnecessary WBCT and its potential complications while avoiding missed injuries.

**Graphical Abstract:**

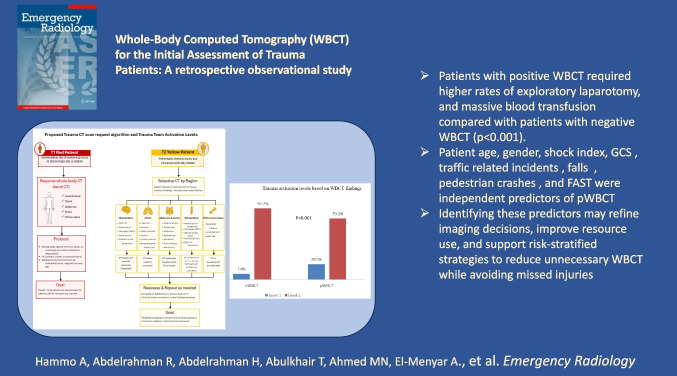

## Introduction

Trauma is a leading cause of death and long-term disability worldwide, particularly among young adults, and contributes substantially to the global burden of morbidity and mortality [1, 2]. Early identification of life-threatening injuries is critical to reducing preventable deaths. International guidelines, such as Advanced Trauma Life Support (ATLS) emphasize the importance of rapid, structured assessment in the initial management of trauma patients [3]. Accordingly, diagnostic tools that support timely and accurate injury detection are central to early trauma care.

Whole-body computed tomography (WBCT) has become an integral part of contemporary trauma evaluation because it enables allowing rapid, single session detection of multi-system injuries in a single imaging session [4, 5]. Compared with conventional diagnostic pathways based on physical examination, focused ultrasound, and plain radiography, WBCT may improve diagnostic efficiency in trauma care by identifying occult injuries that could otherwise be missed [6–8]. However, the key unresolved question is which patients benefit most from WBCT. Comparing patients with positive WBCT findings (pWBCT) versus negative WBCT findings (nWBCT) may help identify the clinical characteristics and outcomes associated with diagnostically meaningful scans.

However, despite its widespread adoption, concerns remain regarding radiation exposure, contrast-related complications, healthcare costs, and the potential overuse of imaging in lower-risk patients [9, 10]. Each whole-body CT scan delivers a considerable radiation dose typically averaging between 20—30 millisieverts (mSv) per scan which is several times higher than standard diagnostic imaging. Moreover, because a substantial proportion of WBCT examinations in trauma settings may be negative (up to 42%) [11], indiscriminate use may expose patients to avoidable risks while increasing resource utilization. These concerns sustain the ongoing debate regarding optimal patient selection for WBCT.

Previous studies have primarily compared WBCT with selective imaging strategies, reporting conflicting results regarding survival benefit and clinical impact. Some observational studies have suggested improved outcomes with WBCT [12–14]. In contrast, randomized controlled trials such as the REACT-2 trial did not demonstrate a significant reduction in mortality [7]. These discrepancies highlight a critical gap, particularly regarding patient level predictors of benefit. The key question is not simply whether WBCT should be used, but which patients are most likely to derive meaningful diagnostic and clinical benefit once it is performed.

Most existing literature has focused on comparisons between WBCT and selective imaging strategies [15]. Far less attention has been given to differences between patients with positive versus negative WBCT findings. This study uniquely investigates the clinical differences between trauma patients with positive versus negative WBCT. A pWBCT defined as the identification of one or more acute traumatic injuries on WBCT, excluding incidental or chronic findings, and including injuries that were clinically significant or resulted in a change in patient management, require intervention, or meet predefined injury severity thresholds [16]. By directly comparing patients based on scan outcomes rather than imaging pathway, this approach addresses an important knowledge gap in understanding patterns of WBCT findings and identifying characteristics associated with clinically significant findings. Such evidence may inform more selective imaging criteria pathways, minimize unnecessary radiation exposure, and improve trauma workflow efficiency. Therefore, we aim to evaluate the clinical characteristics, injury severity, independent predictors of positive WBCT findings, and to compare secondary outcomes including ICU admission, mechanical ventilation duration, ICU and hospital length of stay, and in-hospital mortality between pWBCT and nWBCT groups. These findings may help improve imaging decision-making, refining triage strategies, and optimize patient outcomes.

## Methods

### Study design and setting

This was a retrospective single-center observational study conducted at the Hamad Trauma Center, the national Level I trauma center in Qatar. The center receives approximately 2,200–2,400 trauma admissions annually, of which nearly 30% are major trauma cases (Injury Severity Score [ISS] > 16). Hospitalized trauma patients (≥ 14 years as per our local trauma system agreement for treatment of adult trauma patients) who underwent WBCT performed in our emergency department (ED) between January 2021 and December 2022 were included. Relevant data were extracted from the Qatar National Trauma Registry, which submits quarterly reports to the American College of Surgeons Trauma Quality Improvement Program (ACS-TQIP) and undergoes regular internal and external validation. WBCT was progressively incorporated into trauma management protocols and became standard practice for major trauma cases in 2012. In 2014, the Qatar Trauma System received the Trauma Distinction Award from Accreditation Canada International [17]. At this center, trauma care follows ATLS guidelines, with trauma team activation based on institutional policy.

Patients were excluded if they did not undergo WBCT imaging at presentation, or if they were pregnant at the time of trauma evaluation. Additionally, patients with incomplete data, those who were brought in dead or died upon hospital arrival, transfer patients and inter-facility referrals were excluded from the analysis.

Information extracted for each patient included demographics (age and gender), mechanism of injury, trauma team activation level, admission and discharge dates, vital signs, shock index, Glasgow Coma Score (GCS) score at emergency department, Injury Severity Score (ISS), associated injuries, missed injuries, Abbreviated Injury Scale (AIS) scores, Revised Trauma Score (RTS), Trauma and Injury Severity Score (TRISS), number of repeat CT scans performed for different anatomical regions, Focused Assessment with Sonography in Trauma (FAST) findings, intubation, exploratory laparotomy, massive blood transfusion, ICU admission, ICU length of stay, ventilatory days, total hospital length of stay and in-hospital mortality.

**Tertiary trauma survey** (TTS) consisted of a structured head-to-toe re-examination with review of laboratory and imaging findings, performed by trauma physicians within the first 24 h of admission, typically on the following morning. The final WBCT radiology report was reviewed during TTS. Imaging was re-evaluated only when clinical findings were discordant with the reported findings, with radiology consultation when required.

**Missed injuries** were categorized as type 1 (when identified after the initial assessment but before discharge), and type 2 (injuries missed during both the initial and TTS but identified after hospital discharge).

**WBCT** was defined as the unenhanced head CT followed by contrast-enhanced CT of the chest, abdomen, pelvis, and the complete spine (extremities are not included in the WBCT scope) [18, 19].

A **pWBCT** was defined as the identification of one or more acute traumatic injuries on WBCT, excluding incidental or chronic findings, and including injuries that were clinically significant or resulted in a change in patient management, require intervention, or meet predefined injury severity thresholds [16]. Conversely, a **nWBCT** was defined as the absence of acute traumatic injury across all evaluated regions from head to pelvis [20]. Upper and lower extremity injuries are not indicative of pWBCT, in addition to soft tissue of the face, neck, and trunk.

The **ISS** was calculated as the sum of the squares of the highest AIS code in each of the three most severely injured ISS body regions; it ranges from 1 to 75 |(mild 0—8, moderate 9—15; severe 16—25 and profound > 25). The **AIS** classifies each injury by body region on a six point scale (minor 1, moderate 2, and severe ≥ 3).

**Fig. 1:**
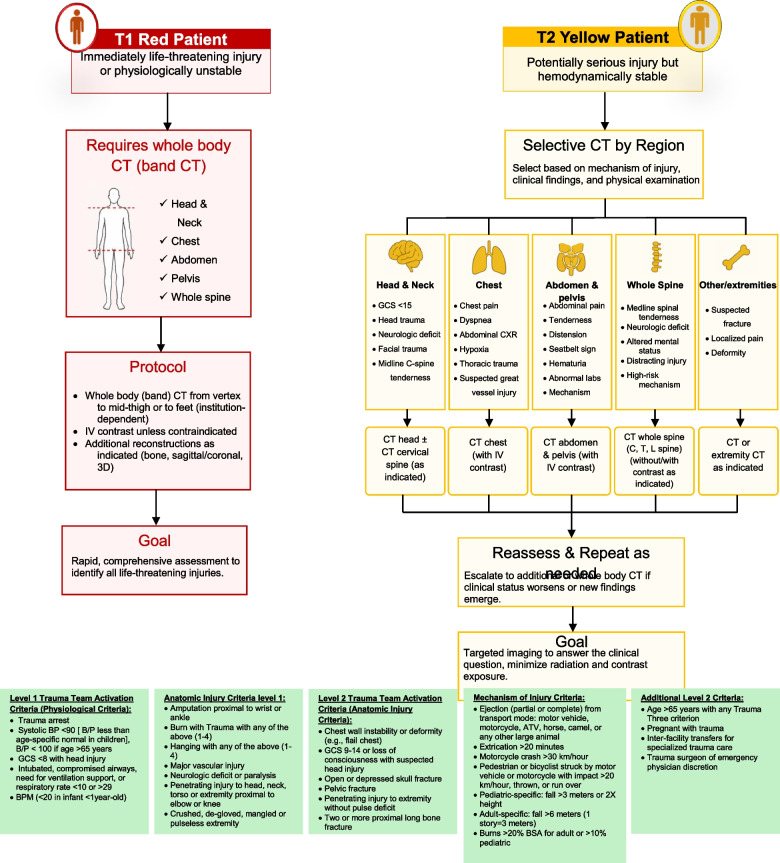
Proposed Trauma CT scan request algorithm and Trauma Team Activation Levels

At our institution, trauma patients are classified into three activation levels (TTA-1, TTA-2, and TTA-3) based on injury severity and predefined physiological/anatomical criteria [21] (Fig. 1).

The primary outcome of this study was to identify factors associated with pWBCT compared with nWBCT findings.

### Imaging protocols

All CT imaging was performed using 128-slice CT scanners (Somatom Drive and Somatom Definition Edge, Siemens Healthineers). Imaging protocols were standardized for trauma patients and included both WBCT and region-specific CT studies, when clinically indicated. Details regarding contrast dose, effective radiation exposure, and imaging acquisition times for each protocol are summarized in Table 1.

**Table 1:** Computed tomography (CT) Imaging Protocols at Hamad Trauma Center

Protocol	Contrast Dose	Effective Radiation Dose (mSv)	Imaging Time (min)
Whole-body CT scan (WBCT)			
Trauma protocol 1 (T1)	Standard IV contrast	60–70	7
T1 + CT carotid angiogram	+ 12–18 ml for carotid	80–90	9
T1 + CT cystogram	+ 7–12 ml for cystogram	65–80	12
Trauma protocol 2 (T2)	Standard IV contrast	45–60	7
Individual CT Studies			
Head/cervical spine	None	10–12	2
Thorax	60–80 ml	6–12	3
Abdomen/pelvis	110–120 ml	8–14	3
CT carotid angiogram	12–18 ml	12–18	2
CT cystogram	7–12 ml	7–12	2

### Statistical analysis

Data were expressed as frequencies and percentages for categorical variables and as mean ± standard deviation (SD) or median (range) for continuous variables, as appropriate. Comparative analyses were performed based on pWBCT and nWBCT groups. The chi-square test was used for the analysis of differences in categorical variables, and Fisher’s exact test was applied when expected cell values were < 5. Continuous variables were compared using Student’s t-test for normally distributed data and the Mann–Whitney U test for non-parametric data. Multivariable logistic regression analysis was performed to determine the predictors of pWBCT and data were expressed as odds ratio with 95% confidence intervals. The most relevant variables were used included the patient age, gender, shock index, GCS, trauma activation level, mechanism of injury and FAST results. A two-tailed *p* < 0.05 was considered statistically significant. Data analysis was carried out using the Statistical Package for the Social Sciences version 26.0 (SPSS Inc., Chicago, Illinois, USA).

## Results

**Fig. 2:**
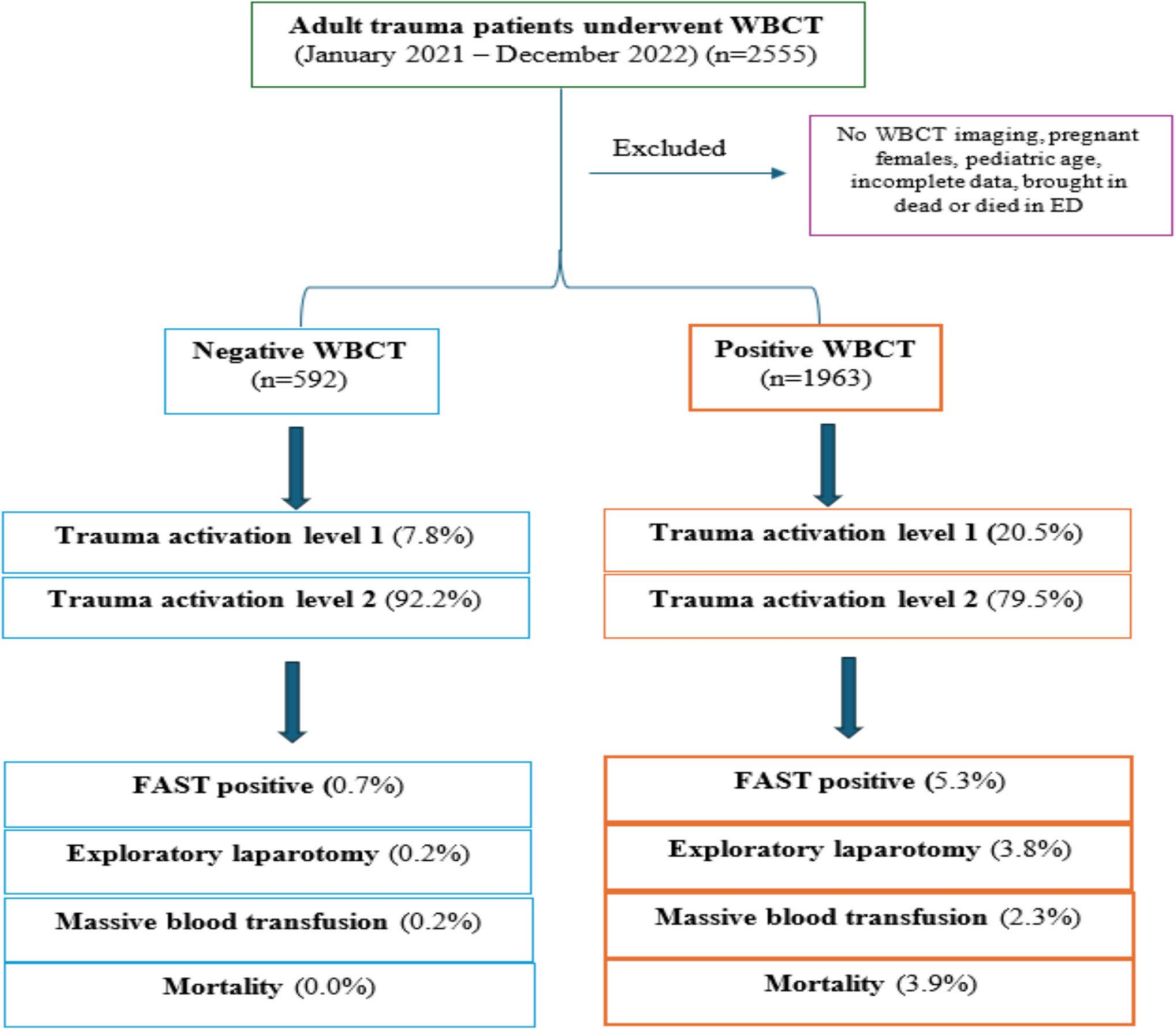
Study design flowchart

During the study period, a total of 3600 trauma patients were admitted to the Hamad Trauma Center, of whom 2555 patients underwent WBCT (71%). Of these, 1,963 patients (76.8%) had pWBCT and 592 (23.2%) had nWBCT (Fig. 2, Table 2). The mean age of the cohort was 34.9 ± 13.5 years, with patients in the pWBCT group being significantly older than those in the nWBCT group (35.6 vs 32.7 years, *p* = 0.001) and most patients were male (90.6%).

**Table 2:** Comparison of demographics, and clinical characteristics, of trauma patients based on WBCT scan findings (January 2021- December 2022)

	Overall *n* = 2555	nWBCT (*n* = 592; 23.2%)	pWBCT (*n* = 1963; 76.8%)	Odd ratio (95% CI)	*P* value
Age (*n* = 2529)*	33 (25—42)	30 (25—39)	34 (26—43)	-	0.001
Males	2315 (90.6%)	540 (91.2%)	1775 (90.4%)	0.90 (0.65—1.25)	0.56
Mechanism of Injury					0.001 for all
Traffic-related accidents	1300 (51.4%)	339 (57.3%)	961 (49.0%)	-	
Fall from height	652 (25.8%)	102 (17.2%)	550 (28.0%)		
Pedestrian	230 (9.1%)	40 (6.8%)	190 (9.7%)		
ATV crashes	134 (5.3%)	21 (3.5%)	73 (3.7%)		
Fall of heavy object	94 (3.7%)	42 (7.1%)	92 (4.7%)		
Assault	43 (1.7%)	14 (2.4%)	29 (1.5%)		
Others	102 (4.0%)	34 (5.7%)	68 (3.5%)		
Shock index	0.66 (0.58—0.78)	0.65 (0.57—0.74)	0.67 (0.58—0.79)	-	0.001
Glasgow Coma score	15 (15—15)	15 (15—15)	15 (15—15)	-	0.001
Injury severity score	10 (5—17)	5 (4—10)	14 (9—21)	-	0.001
Regional injuries					
Head	699 (27.4%)	11 (1.9%)	688 (35.0%)	28.50 (15.58—52.12)	0.001
Chest	911 (35.7%)	1 (0.2%)	910 (46.4%)	510.74 (71.68—3639.08)	0.001
Abdomen	359 (14.1%)	0 (0.0%)	359 (18.3%)	-	0.001
Cervical Spine	222 (8.7%)	4 (0.7%)	218 (11.1%)	18.36 (6.80—49.58)	0.001
Thoracic spine	310 (12.1%)	0 (0.0%)	310 (15.8%)	-	0.001
Lumbar spine	405 (15.9%)	0 (0.0%)	405 (20.6%)	-	0.001
Associated injuries outside the WBCT scope					
Lower limb	1009 (39.5%)	308 (52.0%)	701 (35.7%)	0.51 (0.42—0.61)	0.001
Upper limb	801 (31.4%)	204 (34.5%)	597 (30.4%)	0.83 (0.68—1.01)	0.06
Acute kidney injury	11 (0.4%)	2 (0.3%)	9 (0.5%)	1.35 (0.29—6.30)	0.69
Missed injury	10 (0.39%)	6 (1.01%)	4(0.20%)	-	

*nWBCT* negative WBCT, *pWBCT* positive WBCT, *95% CI* 95% confidence interval; median 95% confidence interval

**Fig. 3:**
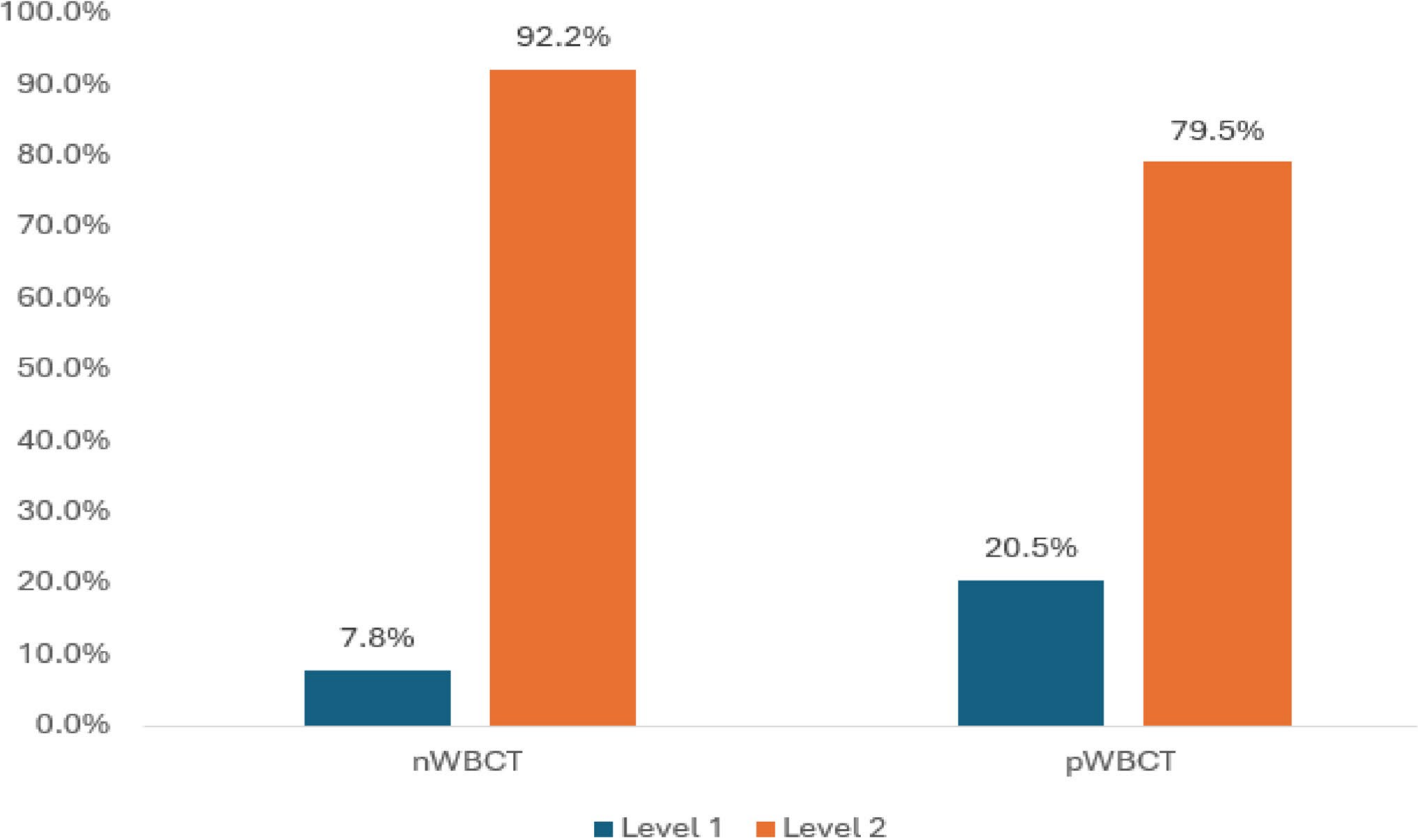
Trauma activation Team Level and WBCT results

Traffic-related incidents were the most frequent mechanism of injury (51.4%), followed by falls from height (25.8%) and pedestrian injuries (9.1%). Falls from height (28.0%) and pedestrian injuries (9.7%) were significantly higher in the pWBCT group. In contrast, cases with traffic-related incidents (57.3%) and fall of heavy objects (7.1%) were more frequent in nWBCT (*p* = 0.001). Most patients were classified as Level 2 trauma activations (82.2%), followed by Level 1 activations (17.4%). Patients in the pWBCT group were more likely to have Level 1 trauma activations (20.5% vs 7.8%; *p* = 0.001), and the nWBCT group had predominantly Level 2 activations (92.2% vs 79.5%; *p* = 0.001) (Fig. 3).

In the ED, the pWBCT group had a significantly higher shock index (0.72 ± 0.24 vs. 0.67 ± 0.16; *p* = 0.001) compared with the nWBCT group. The overall mean ISS was 13.1 ± 9.7, with significantly higher scores in the pWBCT group (15.1 ± 9.9 vs 6.4 ± 4.1; *p* = 0.001) compared with the nWBCT group. Patients in the pWBCT group were more likely to sustain injuries involving the head (*p* = 0.001), chest (*p* = 0.001), abdomen (*p* = 0.001), and spine (*p* = 0.001). The rate of missed injuries was very low in the overall cohort (*n* = 10; 0.39%), but it was higher in the nWBCT group (1.01% vs 0.20%).Table 3 shows the WBCT status across the four ISS categories and the rationale for nWBCT in each category.

**Table 3:** WBCT among the four ISS categories

	Mild ISS (*n* = 812)	Moderate ISS (*n* = 961)	Severe ISS (*n* = 454)	Profound (*n* = 321)
nWBCT	365	207	4	0
pWBCT	447	754	450	321
Explanation for nWBCT	**363** extremity injuries **1** brachial injury, **1** concussion (given AIS)	**201** extremity injuries **2** brachial injury **2** asphyxia **1** sacral nerve contusion by MRI **1** intraoral injury	**3** extremity injury **1** MRI identified spinal cord injury that was not reported on the CT	

ISS categories were defined as mild (0–8), moderate (9–15), severe (16–25), and profound (> 25). ISS for a single region = AIS squared; ISS for 2 regions = AIS squared + AIS squared; ISS for 3 or more regions = Squared AIS of the most severely injured 3 regions

Table 4 summarizes the injury characteristics and outcomes based on WBCT findings. Patients in the pWBCT group had significantly higher head AIS scores ≥ 2 (97.1% vs. 27.3%; *p* = 0.001) and lower Revised Trauma Score (7.2 ± 1.4 vs. 7.7 ± 0.6; *p* = 0.001), and lower TRISS (0.932 vs 0.990, *p* = 0.001) compared with the nWBCT group. The frequency of repeat CT scans by anatomical region did not differ significantly between the two groups, except for CT head which was significantly higher in the pWBCT group (*p* = 0.007). Table 5 provides a case-wise description of patients with head injuries who underwent multiple head CT scans. A positive FAST examination was higher in the pWBCT group (5.3% vs 0.7%, *p* = 0.001), as compared to the nWBCT group. Patients with pWBCT findings required more aggressive interventions, including a significantly higher rate of intubation (*p* = 0.001), exploratory laparotomy (*p* = 0.001), and need for massive blood transfusion (*p* = 0.001).

**Table 4:** Comparison of injury characteristics and outcome of trauma patients based on WBCT scan findings

	Overall *n* = 2555	nWBCT (23.2%)	pWBCT (76.8%)	Odd ratio (95% CI)	P value
Head AIS ≥ 2 (*n* = 699)	671 (96.0%)	3 (27.3%)	668 (97.1%)	89.0 (21.9—360.9)	0.001
Chest AIS ≥ 2 (*n* = 911)	826 (90.7%)	1 (100%)	825 (90.7%)	-	0.74
Abdomen AIS ≥ 2 (*n* = 359)	341 (95.0%)	0 (0.0%)	341 (95.0%)	-	-
Revised trauma score	7.8 (7.8—7.8)	7.8 (7.8—7.8)	7.8 (7.8—7.8)	-	0.001
Trauma and Injury Severity Score (TRISS	0.992 (0.976—0.995)	0.995 (0.993—0.995)	0.991 (0.969—0.994	-	0.001
Number of repeat CT scans					
Head	1 (1—29)	1 (1—1)	1 (1—29)	-	0.007
Chest	1 (1—5)	1 (1—2)	1 (1—5)	-	0.58
Abdomen	1 (1—5)	1 (1—1)	1 (1—5)	-	0.36
Cervical Spine	1 (1—2)	1 (1—2)	1 (1—2)	-	0.52
Thoracic Spine	1 (1—1)	1 (1—1)	1 (1—1)	-	1.00
Lumbar Spine	1 (1—2)	1 (1—1)	1 (1—2)	-	1.00
FAST done:	2531 (99.1%)	591 (99.8%)	1940 (98.8%)	0.14 (0.01—1.05)	0.02
FAST- Negative	2161 (93.9%)	526 (98.0%)	1635 (92.6%)		0.001
FAST- Positive	98 (4.3%)	4 (0.7%)	94 (5.3%)	7.56 (2.76—20.66)	
Intubation	479 (18.7%)	42 (7.1%)	437 (22.3%)	3.75 (2.69—5.22)	0.001
Exploratory laparotomy	76 (3.0%)	1 (0.2%)	75 (3.8%)	23.4 (3.2—169.2)	0.001
Massive blood transfusion	47 (1.8%)	1 (0.2%)	46 (2.3%)	14.18 (1.95—103.0)	0.001
ICU admission	936 (36.6%)	81 (13.7%)	855 (43.6%)	4.86 (3.78—6.25)	0.001
ICU length of stay	3 (2—7)	2 (1—2)	3 (2—8)	-	0.001
Ventilatory days	2 (1—9)	1 (1—1)	3 (1—9)	-	0.001
Hospital length of stay	5 (2—12)	3 (1—9)	5 (2—13)	-	0.001
Mortality	77 (3.0%)	0 (0.0%)	77 (3.9%)	-	0.001

**Table 5:** Description of head injury cases who had multiple head CT scans

Case	Age (yrs)	ISS	Head AIS	Number of repeat head CT scans	Number of interventions	**Non-CT head	Total radiation dose	Hospital LOS	GCS at discharge	Reason
1	46	26	5*	9	4	3	90 mSv	49	GCS9T	Seizure + F/U + ICP + rebleed
2	30	30	5	8	4	3	80 mSv	291	GCS9	ICP + F/U + Seizure
3	21	50	5	7	2	1	70 mSv	86	GCS9T	F/U + Hygroma
4	34	19	3	7	0	0	70 mSv	26	GCS15	Rebleed + F/U
5	22	33	5#*	11	4	1	110 mSv	122	GCS9T	ICP + F/U + seizure
6	24	33	5#	29	4	0	290 mSv	174	GCS7T	ICP + GCS
7	27	26	5	9	5	1	90 mSv	92	GCS9T	ICP + F/U + GCS
8	21	43	5#	12	3	0	120 mSv	80	GCS8T	F/U + GCS
9	19	38	5	8	3	2	80 mSv	65	GCS6T	GCS + F/U + hygroma
10	21	21	4	15	4	2	150 mSv	160	GCS9T	Seizure + GCS + rebleed
11	23	24	4	11	2	0	110 mSv	92	GCS13	GCS + F/U
12	51	25	5@	5	2	0	50 mSv	22	Death	Seizure + F/U
13	20	17	4#	11	3	3	110 mSv	106	GCS12	GCS + F/U
14	73	29	4	8	1	1	80 mSv	55	GCS9T	F/U
15	22	10	3	8	2	1	80 mSv	25	GCS14	GCS + F/U

Interventions: ICP, Craniectomy, Craniotomy, EVD, Cranioplasty, Hydrocephalus, VP shunt; *PE; #VAP; @mortality; *F/U* follow-up, *GCS* Glasgow Coma Score, *AIS* abbreviated injury score, *ICP* Intracranial pressure, *ISS* Injury Severity Score; **mostly to rule out pulmonary embolism CTC (non-head) follow-up

### Outcomes

The pWBCT group experienced significantly worse clinical outcomes, including higher rates of ICU admission (*p* = 0.001), longer mechanical ventilation duration (*p* = 0.001), and prolonged ICU length of stay (*p* = 0.001) and hospital stay (*p* = 0.001). Mortality was also significantly higher in the pWBCT group compared with the nWBCT group (3.9% vs. 0.0%; *p* = 0.001).

### Predictors of pWBCT

Multivariable logistic regression analysis demonstrated that increasing age (aOR 1.013; 95% CI 1.004—1.022; *p* = 0.003), male gender (aOR 0.687; 95% CI 0.475—0.995; *p* = 0.047), shock index (aOR 1.990; 95% CI 1.045—3.789; *p* = 0.036), lower GCS at ED (aOR 0.852; 95% CI 0.804—0.904; *p* = 0.001), mechanisms of injury including traffic-related incidents (aOR 1.464; 95% CI 1.065—2.012; *p* = 0.019), falls from height (aOR 2.545; 95% CI 1.750—3.701; *p* = 0.001), pedestrian crashes (aOR 1.620; 95% CI 1.013—2.588; *p* = 0.044), and a positive FAST examination (aOR 5.977; 95% CI 2.149—16.623; *p* = 0.001) were independent predictors of pWBCT findings in trauma patients (Table 6).

**Table 6:** Multivariable regression analysis for the predictors of pWBCT

Variable	Odds Ratio	95% Confidence Interval		P-value
Age in years	1.013	1.004	1.022	0.003
Gender Males	0.687	0.475	0.995	0.047
Shock index at the ED	1.990	1.045	3.789	0.036
Glasgow Coma Scale at the ED	0.852	0.804	0.904	0.001
Trauma level 1 activation	0.952	0.609	1.487	0.828
Mechanism of injury				
Traffic related incidents	1.464	1.065	2.012	0.019
Fall from height	2.545	1.750	3.701	0.001
Pedestrian	1.620	1.013	2.588	0.044
Fall of heavy object	1.783	0.957	3.324	0.069
Positive FAST	5.977	2.149	16.623	0.001

*ED* emergency department, *FAST* Focused Assessment with Sonography in Trauma

## Discussion

This study provides a detailed characterization of trauma patients with positive versus negative WBCT at a national Level I trauma center. The findings demonstrate that pWBCT represents a distinctly more severely injured population, characterized by higher trauma activation levels, elevated shock index on presentation, lower GCS scores, and mechanisms of injury associated with high-impact energy. Also, patients with pWBCT had significantly higher ISS and a greater burden of multiple anatomical regions. The increased need for advanced life-saving interventions such as intubation, exploratory laparotomy, and massive transfusion. These results indicate that pWBCT primarily reflect patients with greater anatomical injury severity and physiological derangement rather than being an independent predictor of adverse outcomes. Our observations are consistent with previous studies [22] and contribute to the ongoing debate regarding the use of WBCT in trauma management [23].

The reported proportion of nWBCT scans in trauma patients varies widely in the literature, ranging from 7% to as high as 57% [20]. In our cohort, approximately three-quarters of patients who underwent WBCT demonstrated positive radiological findings, while 23.2% showed negative WBCT findings. Consistent with these observations, Bolbol et al. [24] reported that 37.3% exhibited single-organ injuries, 32.7% demonstrated polytrauma involvement, and nWBCT findings were observed in 30% of cases. These differences are likely driven by variation in case mix, trauma activation thresholds, institutional imaging practices, and regional injury epidemiology. In addition, residual confounding inherent to observational and registry-based studies limits causal inference when comparing outcomes across trauma systems.

In contrast, a study from Kuwait reported predominantly nWBCT results, with 75.8% of polytrauma patients showing no trauma-related radiological abnormalities and only 24.2% demonstrating positive findings [25]. These variations likely reflect differences in patient selection criteria, injury epidemiology, and institutional WBCT utilization protocols across trauma systems. Importantly, understanding the characteristics of patients with nWBCT is clinically relevant, as this group demonstrats lower injury severity, fewer interventions, and shorter hospital stays, suggesting that refining selection criteria may reduce unnecessary imaging while maintaining patient safety.

In the present study chest injuries (35.7%) were most prevalent followed by head injuries (27.4%). These frequencies reflect the overall injury distribution in the study cohort and should not be interpreted as determinants of pWBCT status. Because standard WBCT was defined as head-to-pelvis imaging, upper and lower extremity injuries were recorded separately in the trauma registry and were not included in the classification of pWBCT. Patients with pWBCT findings were significantly more likely to sustain multisystem injuries. These injury patterns are broadly comparable with findings from a recent systematic review, reporting head, chest, abdominal, and spinal as the most frequently detected injuries among trauma patients undergoing WBCT [19]. These observations are consistent with prior reports, demonstrating that WBCT is preferentially used in patients with higher injury severity than those managed with selective imaging strategies [26].

Our analysis identified independent predictors of pWBCT, including increasing age, higher shock index, lower GCS at presentation, high-risk mechanisms of injury, and a positive FAST examination. These factors represent readily available clinical indicators [27] that may help trauma teams identify patients most likely to benefit from comprehensive imaging. It is important to note that the observed association between positive WBCT findings and higher mortality primarily reflects the underlying injury severity of this group. Rather than supporting indiscriminate WBCT use, our findings reinforce the importance of clinically guided imaging decisions based on physiological and injury mechanism-based risk stratification. The predictors identified in this study may also have practical implications for future decision-support strategies. Variables that are immediately available during initial trauma assessment, such as age, SI, GCS, mechanism of injury, and FAST findings, could be integrated into a clinical prediction tool (e.g., risk score or nomogram) to estimate the pre-test probability of pWBCT findings. Such an approach may help standardize imaging decisions, prioritize WBCT for higher-risk patients, and reduce unnecessary imaging in lower-risk cases.

An interesting finding in our study was the significantly higher proportion of positive FAST examinations in the pWBCT group. This may be explained by the diagnostic role of FAST, which preferentially detects free intraperitoneal or pericardial fluid in patients with clinically significant hemorrhage [28]; such patients may proceed directly to operative or interventional management based on FAST findings, thereby reducing the likelihood of subsequent WBCT confirmation. This highlights the complex interaction between bedside assessment tools and advanced imaging strategies during trauma resuscitation.

In our study, pWBCT findings were associated with more severe head injuries and worse trauma severity indices. In clinical practice, patients with suspected multisystem or severe injuries are more likely to undergo comprehensive imaging [29], introducing an inherent selection bias that must be considered when interpreting differences in outcomes.

Table 7 summarizes published studies comparing clinical outcomes of WBCT versus selective imaging in trauma patients [7, 8, 14, 26, 30–34]. Analyses from the German Trauma Register have reported improved survival among patients undergoing WBCT [31], including those who are hemodynamically stable [8]. However, these studies acknowledged the persistent influence of residual confounding, particularly injury severity, prehospital triage, and institutional practices. The current study did not evaluate time-related outcomes such as imaging duration or emergency department length of stay, nor did it compare WBCT with selective imaging strategies. Consequently, our findings cannot be directly interpreted as evidence for or against the diagnostic or prognostic superiority of WBCT.

**Table 7:** Studies comparing outcomes of WBCT vs selective imaging in trauma

Study	Design/Setting	n	Primary outcome	Findings
Sierink et al. 2016 [7]	Multicentre RCT	1,403	In-hospital mortality	No difference (16% vs 16%); faster imaging
Huber-Wagner 2009 [31]	Multicentre registry (TraumaRegister DGU)	4,621	Survival (SMR/TRISS, RISC)	Improved survival with WBCT
Huber-Wagner 2013 [8]	Multicentre registry (shock included)	16,719	Mortality (overall & by shock)	Lower mortality with WBCT overall and in shock
Jiang 2014 [32]	Meta-analysis (11 studies)	26,371	Mortality	Reduced mortality (OR 0.66); shorter ED time
Hajibandeh 2015 [30]	Systematic review/meta-analysis	24,468	Mortality	Reduced mortality; heterogeneity present
Stengel et al. 2012 [14]	Retrospective cohort	982	Diagnostic accuracy	Initial WBCT showed high diagnostic accuracy, (sensitivities: 79.6–86.7% and specificities: 97.5–99.8%) across injury regions
Mohamed et al. 2021 [34]	Retrospective cohort	481	LOS, repeat CTs, mortality	Shorter LOS, fewer repeat CTs; no mortality difference
Caputo et al. 2014 [26]	Systematic review/meta-analysis	25,782	Mortality	WBCT associated with lower mortality (OR 0.75)
Tang et al. [33]	Systematic review/meta-analysis	14,908	Mortality	WBCTs are not significantly associated with a reduced risk of mortality (OR: 0.88)

*RCT* randomized controlled trial, *WBCT* whole body CT scan, *ED* emergency department, *OR* Od ratio, *LOS* length of stay

Moreover, the REACT-2 randomized controlled trial [7] demonstrated that immediate WBCT significantly reduced imaging time compared with selective imaging, but this efficiency did not translate into a mortality benefit. This suggests that while WBCT may improve workflow and diagnostic speed, survival outcomes are more strongly influenced by injury severity, timely interventions, and overall quality of trauma care rather than imaging strategy alone. Similarly, meta-analyses [30, 35] have suggested that WBCT may be associated with reduced emergency department times and mortality; however, the predominance of observational studies in these analyses limits causal inference, and the findings may therefore be influenced by selection bias. Importantly, our study was not designed to compare WBCT with selective imaging strategies or evaluate imaging time metrics; therefore, the results should be interpreted within the context of clinical characterization rather than imaging effectiveness.

Other international comparisons support a nuanced perspective. A UK cohort study involving elderly trauma patients reported shorter hospital stays and fewer repeat imaging studies among those undergoing WBCT, although mortality rates were unchanged [34]. Conversely, an Australian study focusing on hemodynamically stable trauma patients highlighted concerns regarding increased radiation exposure and the detection of incidental findings, raising the possibility of WBCT over-utilization in a lower-acuity population [36]. Prior data showed that WBCT was more frequently employed in severely injured and physiologically unstable patients, with apparent survival benefit associated with WBCT [36–38].

Beyond describing clinical differences, the proportion of nWBCT scans observed in our cohort raises important considerations regarding imaging utilization. Although negative findings do not necessarily imply inappropriate imaging, they may reflect a subset of patients who underwent comprehensive CT despite having lower injury severity and limited need for intervention. WBCT is associated with substantial radiation exposure, increased healthcare costs, and potential downstream consequences, including incidental findings and unnecessary follow-up investigations [11].

A significant strength of this study is its large sample size from a high-volume national Level I trauma center, which enhances the generalizability of findings to similar tertiary trauma systems. Additionally, standardized imaging protocols and internal validation of data collection improve the reliability and consistency of the reported results. Nevertheless, further external validation is needed to strengthen the applicability of these results. Clinicians should consider using clinical predictors to guide selective use of whole-body CT imaging in trauma patients, rather than relying on routine WBCT for all. Integrating these readily available clinical indicators into trauma workflows can help prioritize WBCT for patients most likely to benefit, reduce unnecessary imaging in lower-risk cases, and improve resource utilization without compromising early injury detection. As there is no standardized international algorithm for the routine use of WBCT versus selective CT, a standardized protocol could be of value in trauma patients. This will balance the risks of complications (radiation and contrast), cost, and incidental findings versus the risk of missed injuries, repeat imaging, and delays in triage and on-time treatment [39]. We have proposed an algorithm for the indications of WBCT in trauma that can be validated in future research (Fig. 1).

### Limitations

First, the retrospective design introduces inherent selection bias, as WBCT was more frequently employed in severely injured patients. Second, prehospital variables and comorbidities were not fully accounted for, potentially influencing imaging decisions and outcomes. Third, radiation exposure was estimated using standard protocol doses [40] rather than patient-specific measurements, and long-term risks, such as radiation-induced malignancy, could not be assessed. Fourth, incidental findings detected on WBCT and their clinical and economic consequences were not analyzed. Fifth, the exclusion of patients brought in dead or who died upon hospital arrival may have introduced survivor bias and limited representation of the most severely injured trauma population. Sixth, WBCT does not include assessment of upper or lower extremity injuries (both are outside the scope of pWBCT), therefore selective imaging should be requested based on the clinical judgment and surveys to minimize the potential missed injuries. Finally, a cost analysis was not performed, limiting the evaluation of WBCT’s financial impact.

In **conclusion**, the distinction between pWBCT and nWBCT patients provides valuable insight into trauma workflow and resource utilization. Patients with positive WBCT findings represent a clinically distinct subgroup characterized by greater injury severity, greater physiological compromise, and a higher need for critical interventions. Also, the proportion of nWBCT scans observed in the study cohort raises important considerations regarding imaging utilization. Therefore, identifying predictors of positive scans may assist trauma teams in refining imaging decision-making and optimizing resource utilization. These factors represent readily available clinical indicators that may help trauma teams identify patients most likely to benefit from comprehensive imaging. Future prospective studies should evaluate whether clinically guided risk-stratification models can safely reduce the proportion of negative WBCT examinations without compromising early injury detection.

## Data Availability

Data will be available on reasonable request from the PI after approval of the medical research center, Doha, Qatar.
